# The Unethical Texas Heartbeat Law

**DOI:** 10.1002/pd.6136

**Published:** 2022-04-09

**Authors:** Casey Michelle Haining, Louise Anne Keogh, Julian Savulescu

**Affiliations:** ^1^ Centre for Health Equity Melbourne School of Population and Global Health University of Melbourne Melbourne Victoria Australia; ^2^ Oxford Uehiro Centre for Practical Ethics University of Oxford Oxford UK; ^3^ Murdoch Children's Research Institute Melbourne Victoria Australia; ^4^ Melbourne Law School University of Melbourne Melbourne Victoria Australia

## Abstract

What is already known?

- The Texas Heartbeat Act, which has been in effect since September 2021, prohibits abortions once a ‘fetal heartbeat’ is detected, except in emergency situations.
- The law significantly limits access to abortion services in Texas, by essentially prohibiting abortions post 6 weeks' gestation.
- The law has been subjected to several legal challenges, none of which have been successful to date.

What does this article add?

- This article provides an overview of some of the ethical concerns the law raises and identifies some of the problems the law creates for women, the health profession and society. The article ultimately argues that the law ought to be quashed.

## 1 INTRODUCTION

The ‘Texas Heartbeat Act’ found in the *Texas Health & Safety Code* (§§ 171.201–171.212), hereafter referred to as ‘the Act’, prohibits abortions once a ‘fetal heartbeat’ is detected, except in emergency situations. Emergency situations are limited to “a life‐threatening physical condition aggravated by, caused by, or arising from a pregnancy that, as certified by a physician, places the woman in danger of death or a serious risk of substantial impairment of a major bodily function unless an abortion is performed” (§171.002). The law makes no exceptions for pregnancies resulting from rape or incest.

What makes the law unique is that it is enforced exclusively by the actions of private citizens bringing civil lawsuits rather than being directly enforced by the State. This means any person, other than an officer or employee of a state or local governmental entity, can bring a case against any person who “performs or induces an abortion” or any person who “aids or abets the performance or inducement of an abortion” once a ‘fetal heartbeat’ is detected (§171.208). While the woman seeking an abortion cannot be sued, the Act's framing is so broad it could potentially impact health professionals, reception staff at a healthcare clinic, family members and friends who counsel the woman, and Uber drivers who drive women to abortion clinics. Furthermore, the person suing does not need to show any connection to those they are suing, and if they are successful, they will receive a minimum of $10,000 (US dollars) and have their legal fees covered.

## 2 THE HISTORY OF THE ACT

To date, 13 US states have enacted ‘heartbeat laws’.^1^ Until now, all these laws have been struck down by state supreme courts and the US Supreme Court.^2^ According to Evans and Narasimhan, US fetal heartbeat bills have increasingly become the anti‐abortion legislative measure of choice, since they were first introduced in 2011.^3^ The legislative models adopted by other states were framed in a way which would allow individuals to sue state officials for enforcing an unconstitutional law because these laws directly challenged the federal protection under *Roe v Wade*, which recognizes the right of a woman to choose to have an abortion up to fetal viability as part of the constitutional right to privacy.^4^ The Texas law, however, adopts a different framing. The ‘clever’ drafting of the Act and privatization of enforcement means that judicial review can be evaded^1^ and state officials are shielded from being sued for violating the constitution, which arguably makes the law more durable and difficult to challenge.^5^, ^6^

The Law was passed in May 2021, and was signed by Texas Governor, Greg Abbott (Republican), who said:

“Our creator endowed us with the right to life and yet millions of children lose their right to life every year because of abortion. In Texas, we work to save those lives.”^7^

The day before the ban came into effect many women raced against the clock to secure an abortion. One abortion clinic reportedly performed 67 abortions in 17 h to beat the new ban.^8^ In a midnight ruling on 1 September, 2021, just prior to when the Act was due to come into force, the US Supreme Court refused to block the legislation, despite appeals from reproductive rights organizations, civil rights organizations, and abortion clinics.^9^ The Supreme Court's 5‐4 decision reflected its newly conservative leaning shaped by former president Donald Trump.^9^ The Supreme Court claimed that it was not ruling on the constitutionality of the Texas law, but was allowing it to go ahead because of complex legal and procedural questions.^9^

The Act came into effect on 1 September, 2021. Following its introduction, there have been several legal challenges; however, no challenge has been successful to date.^10^ In December 2021, the US Supreme Court left the door open for future challenges in lower courts on narrow grounds, which was subsequently shut down by the Texas Supreme Court in March 2022.^11^

## 3 THE RATIONALE BEHIND THE LAW AND THE SIGNIFICANCE OF A ‘HEARTBEAT’

In the Act, fetal heartbeat is defined as “cardiac activity or the steady and repetitive rhythmic contraction of the fetal heart within the gestational sac” (§171.201). Such cardiac activity can be detected as early as 6 weeks.^1^At the core of the Act is the idea that as soon as a fetal heartbeat can be detected, the fetus should be considered a person and afforded rights and protections.^12^ The sound of the heartbeat is thought to define humanity and therefore justifies a protection of rights.^12^ These sentiments are echoed in the legislature findings of the Act:

§171.202 Legislative Findings.

The legislature finds, according to contemporary medical research, that:

(1) fetal heartbeat has become a key medical predictor that an unborn child will reach live birth;
(2) cardiac activity begins at a biologically identifiable moment in time, normally when the fetal heart is formed in the gestational sac;
(3) Texas has compelling interests from the outset of a woman's pregnancy in protecting the health of the woman and the life of the unborn child; and
(4) to make an informed choice about whether to continue her pregnancy, the pregnant woman has a compelling interest in knowing the likelihood of her unborn child surviving to full‐term birth based on the presence of cardiac activity.

However, the use of ‘heartbeat’ in the Act is contested and misleading. Medical and reproductive health experts argue that referring to a ‘heartbeat’ is medically inaccurate, as the embryo does not have a developed heart at 6 weeks' gestation.^13^ The presence of cardiac activity is not equivalent to the presence of a functioning heart or heartbeat, which is defined as the pulsation of the heart.^3^ According to Dr Jen Gunter (Canadian Gynecologist):^14^

“An embryo does not have a heart – at least, not what we understand a human heart to be, with pumping tubes and ventricles. At six weeks, a human embryo throbs, but those tissues have not yet formed an organ, so the pulsing should not be confused with a heartbeat.”

## 4 POSITIVE CONSCIENCE CLAIMS AND DELIBERATE DISOBEDIENCE

Heartbeat laws have been argued to result in “unjustified asymmetry” as they fail to accommodate health professionals “who may find themselves deeply morally opposed to such legislation, believing that they cannot, in good conscience, deny providing an abortion to a woman who requests it, even after a heartbeat is detected”.^15^ Such health professionals believe they have a *positive obligation* to provide abortions for conscience reasons. Indeed, this played out days after the law was introduced, with a Texas Doctor, Dr Alan Braid, publicly admitting in an op‐ed piece in the Washington Post that he violated the Texas law.^16^ He wrote:

“[O]n the morning of Sept. 6, I provided an abortion to a woman who, though still in her first trimester, was beyond the state's new limit. I acted because I had a duty of care to this patient, as I do for all patients, and because she has a fundamental right to receive this care … I fully understood that there could be legal consequences — but I wanted to make sure that Texas didn't get away with its bid to prevent this blatantly unconstitutional law from being tested.”

Dr Braid was subsequently sued by two plaintiffs, neither of whom were anti‐abortion, but both decided to sue so the law’s legality could be tested.^17^

## 5 PROBLEMS WITH THE ACT

In addition to the ethical concerns the Act raises, which is discussed in the next section, the Act is problematic for several other reasons including its ability to: undermine a woman's autonomy to decide whether to proceed with a pregnancy beyond a narrow gestational limit; deny access to an in‐demand and evidence‐based health service; increase the likelihood of self‐managed abortions; lead to intimidation through establishing a vigilante system; and undermine medical expertise.

### 5.1 Narrow gestational limits and undermining women's autonomy

The Act undermines women's autonomy and their right to make decisions about their own sexual and reproductive health, by denying them the ability to make a choice about whether to proceed with a pregnancy, which most women at 6 weeks' gestation are unlikely to be aware of.^1^ Even if a woman is aware of the pregnancy, abortion appointments may not be available for several days or weeks at abortion clinics.^18^ As such, even women who seek care early in pregnancy may not be able to obtain a lawful abortion within the permissible gestational limits due to inadvertent delays.

Six weeks' gestation also means that women are not able to undertake screening tests for fetal abnormalities and are therefore denied the opportunity to make informed medical decisions.

As indicated by a statement provided by the president of the American Society of Human Genetics:

“The law denies women and their families the ability to use health information stemming from human genetics research by prohibiting abortion after detection of a fetal heartbeat … Because many devasting diagnoses can only be determined later in fetal development … [the Texas law] will prevent women and families from using genetic information to inform reproductive decisions.”^19^

While the use of prenatal screening and selective abortion is still viewed by some as being ethically contentious, it is more widely viewed as an essential part of reproductive or procreative autonomy or liberty.^20^ Some commentators have argued that abortion can reduce potential harm to the unborn child, if the pregnancy would result in a child being born with severe structural defects or a severe genetic abnormality. In such cases, abortion is believed to prevent the unnecessary suffering of the child and its family, and is supported by the principle of non‐maleficence.^21^ Gillam argues that in the special circumstance of fetal abnormality, consideration of the quality of life of the child‐to‐be could justify selective abortion when one accounts for the effects of the disability and compares the quality of life of the child‐to‐be with a non‐disabled child.^22^ Similarly, Savulescu argues that there is a moral reason to have a child who is less likely to suffer a genetic condition predicted to reduce that child's health and wellbeing.^23^

Women are also strongly in favor of access to genetic testing and selective abortion. Research carried out with women who underwent abortions due to fetal abnormality found that, although women have a choice in such cases, they typically felt like they had no choice.^24^ The study reported that women would proceed with the abortion out of compassion, in order to spare a child of a life of suffering.^24^ In relation to major fetal abnormalities, research has found that even women who consider themselves to be anti‐choice re‐evaluate their in‐principle opposition to abortion.^25^

### 5.2 Curtailing safe access to an in‐demand evidence‐based health service

Not only does the Texas law undermine women's autonomy, it also limits access to medical care that is evidence‐based and inhibits the delivery of safe, timely and necessary comprehensive care.^26^ As claimed by the President of the American Medical Association:^27^

“This new law is a direct attack on the practice of medicine and patient reproductive health outcomes. As physicians and leaders in medicine, we urge our nation's highest court to take action immediately … [f]ailure to do so places physicians' clinical judgement and patient access to safe care in dire peril.”

Prior to the Act's introduction, an estimated 55,000 abortions were performed each year in Texas.^28^ The number of abortions performed have greatly reduced since the Act's introduction. The first month in which the law was in force saw a 60% decline in abortions performed in Texas.^29^ The impact of the Act is particularly profound for vulnerable and marginalized populations. Indeed, some commentators have argued that the law “disproportionately affects black, Hispanic, and women on low incomes, who already face obstacles to [health care].”^7^ Such a claim is supported by empirical evidence which has found that patients obtaining second‐trimester abortions in Texas were more likely to be black, on low incomes, and required to travel long distances to obtain in‐clinic care.^30^ Given the restrictive nature of the law, the only way women will now be able to access an abortion will be to travel out of Texas. Commentators have predicted that the closure of Texas abortion clinics will increase the average driving distance to a clinic from 19 to 399 km.^31^ Due to financial constraints, many women will be unable to travel, and hence will be denied access to abortion services. Moreover, even if women can afford to travel, there is no guarantee they will be able to secure an abortion in other states. Indeed, neighboring states' reproductive health clinics have experienced a flooding of patients following the commencement of the Act, which has raised concerns that clinics in neighboring states will not be able to absorb all the new patients.^28^

### 5.3 Increased likelihood of self‐managed abortion

The restrictive Texas law may also increase the likelihood of women seeking to self‐manage their abortion rather than seeking medical assistance, resulting in unsafe abortion procedures. The prevalence of unsafe abortion remains the highest in jurisdictions which have the most restrictive abortions laws and/or policies.^32^ Making abortion legal, safe, and accessible does not appreciably increase abortion demand; it reduces clandestine and unsafe abortion procedures and results in legal and safe ones.^32^ Unsafe abortion procedures may result in medical complications and abortion‐related mortality.^32^

Importantly, not all self‐managed abortions will necessarily be unsafe. Contemporary reports of self‐management in the US found that a lot of the self‐managed abortions are not necessarily the product of the use of sharp objects or back‐alley providers, but rather medications such as mifepristone and misoprostol.^33^ This is believed to parallel with the rise of the Internet being the ‘go‐to source for information and services’.^33^ Previous research has found that medical abortion is quite common amongst the Texas population, partly due to the close proximity with the Mexican border, where medical abortion can more easily be obtained due to its large immigration population from Latin America (who tend to be familiar with self‐managed abortion and navigating restrictive abortion laws).^34^

At the time of its introduction, it was predicted that the Texas law would increase the number of women seeking to self‐induce abortions using pills obtained by mail.^31^ Due to such a threat, the Texas Governor signed a separate bill (SB4 bill) on September 17, 2021 which came into effect on December 2, 2021.^35^ The new law narrows the window in which physicians are allowed to give an abortion‐inducing medication to 7 weeks, which also covers medication sent by courier, delivery or mail service. Violations of the law are punishable by up to 2 years in prison and a fine of up to $10,000 (US Dollars).^35^

### 5.4 Establishment of a vigilante system that intimidates

Another issue with the Act is that by enabling private enforcement, it incentivizes strangers to spy on women^36^ and encourages vigilante justice.^37^ Some commentators have expressed concern that the Act could increase the legal risk for health professionals and expose them to frivolous lawsuits,^38^ given the Act essentially creates a pathway for litigation that serves to intimidate and dissuade medical practitioners from providing patients with the medical care they need.^39^ Such apprehension was reflected in a joint statement made by leading American physician groups:

“Physicians must be able to practice medicine that is informed by their years of medical education, training, experience, and the available evidence, freely and without threat of punishment, harassment, or retribution.”^26^

### 5.5 Undermine medical expertise

The overhanging threat of litigation that results from the Act is also problematic as it may discourage health professionals from acting in accordance with clinical standards and undermine their medical expertise. For example, in clinical presentations such as cardiomyopathy, lupus and nephrotic syndrome,^40^ where there is an increased risk of maternal morbidity or morality, but at the time of diagnosis the woman may not be considered to be in imminent danger, health professionals may be reluctant to terminate the pregnancy out of fear that such a decision could be scrutinized in court due to ambiguity around whether such a scenario would fall within the ambit of the medical emergency exception.^40^ Similarly, it has been argued that the law ignores medical conditions that are common indications for termination such as fetal reduction for some cases of severe twin‐twin transfusion syndrome, or pre‐viable premature rupture of membranes when the decision to terminate the pregnancy before the woman is severely ill and at imminent risk of dying from sepsis could be subjected to a legal challenge.^40^ Accordingly, the law's narrow framing, and the degree of ambiguity it creates, has the potential to impinge on clinical decision‐making and compromise the health professionals ability to act in the best interests of the woman.

## 6 ETHICS

According to one proponent, Senator Bryan Hughes:

“The heartbeat is the universal sign of life … [i]f a Texan's heartbeat is detected, his or her life will be protected.”^13^

There are two kinds of termination of pregnancy: (1) medical; (2) social. Medical terminations of pregnancy are performed either to protect the medical interests of the woman or to prevent the fetus going on to live a life which is not in its interests, that is, a life not worth living because of a disorder which is severe. The law gives some consideration of the severe threat to maternal medical interests, but no consideration of termination in the case of severe fetal abnormality. While it is hard to draw the line at which life becomes no longer worth living, there is a general consensus that some fetal abnormalities such as anencephaly, severe epidermolysis bullosa or Lesch‐Nyhan syndrome may make life so devoid of positive experience as to be not worth living.^41^

Social terminations are performed to respect the autonomy of the woman or promote her wider interests or well‐being. At the heart of this law is the vexed question of when a human organism acquires a right to life and special protection in law. The most rigorous analysis of the ethics of abortion is Jeff McMahan's ‘The Ethics of Killing: Problems at the Margins of Life’.^42^ McMahan's view is that a human being does not acquire a moral status until some higher form of consciousness begins after birth.^42^ This entails the permissibility of certain forms of infanticide.^43^

Most liberal legal jurisdictions are based on the view that the fetus does not acquire moral status until birth, or at least the woman's interests and autonomy outweigh any moral status until that point. Such a view of moral status allows early and late abortion. We cannot settle the issue of fetal moral status or the ethics of abortion in this short commentary. What we aim to show in this section is that there is *no plausible ethical account of moral status* that supports the Texas law. We consider 3: (a) consciousness‐based accounts, (b) potentiality accounts; and (c) gradualist accounts.

(a) Consciousness‐based accounts of moral status

The law itself is careful to try to avoid this issue directly, by arguing that the presence of a fetal heartbeat indicates a high chance that the baby will go on to be born alive. But this begs the question around moral status: is being born alive what matters? An anencephalic baby can be born alive, only to die soon after birth. Even if the means were available to keep an anencephalic baby alive indefinitely, most people would argue that such a life should not be prolonged because of the total lack of consciousness, or anything of value in human life.

Modern technology can identify that an embryo is alive and developing. There are more and more predictors of the probability of normal development and birth, such as whole genome analysis and artificial intelligence assisted selection of embryos when in vitro fertilization is performed.^44^ But it is not mere probability of survival that matters; it is something distinctive about human life that accords it special significance and rights.

One way to address when moral status begins is to ask when does it end? If we look to the other end of life – death – we gain one insight into what matters.^45^ Over 50 years ago, most countries moved from a cardiorespiratory definition of death to a brain death definition. It is not the presence of a heartbeat that matters, but the presence of brain function. Indeed, some have even called for a move from whole brain or brainstem death to neocortical death – the complete lack of consciousness. While no country has adopted a neocortical definition of death, many jurisdictions allow the withholding or withdrawing of life‐prolonging medical treatment if a patient is permanently unconscious. As was reasoned in the landmark UK case of *Airedale National Health Service* *Trust v Bland* (which has been widely applied), prolonging life by administering treatment that is futile (such as when a person is unconscious with no chance of improving) cannot be in the patient's best interests, and withholding and withdrawing treatment in such cases would be lawful.^46^

So, if our life, our biography as James Rachels put it,^47^ ends when we become unconscious permanently, it begins when we become conscious. Consciousness depends on the activation of the cortex by thalamocortical connections around 24 weeks after conception.^48^ Indeed, some philosophers such as Jeff McMahan,^42^ Peter Singer^49^ and Michael Tooley^50^ have argued, it is not mere consciousness that matters, but high levels of consciousness such as self‐consciousness or rational/moral capacities, which do not commence until after birth. This would allow late abortion (and infanticide).^43^

(b) Potentiality accounts of moral status

The main argument against such views of moral status which allow abortion is the argument that the embryo and fetus prior to consciousness have the capacity or potential to lead a full human life with self‐consciousness and advanced cognitive capacities. There are arguments for and against potentiality as a ground for moral status.^51^, ^52^ The most prominent argument against it is that a potential X does not have the same rights as an actual X: a potential King of England does not have the same rights as an actual King. But even if the potentiality argument were to succeed, it would not support the Texas law as the embryo from the moment of conception has the potential to be a person. Potentiality might justify a complete ban, but not a heartbeat law.

The approach taken in the legislation is to argue that the probability of personhood increases with the presence of a heartbeat. But the mere increase in chance of being born alive does not affect potentiality or moral status. We do not say that a person with terminal condition that cannot be treated has no right to life. They do have a right to life – we just cannot satisfy it. They have moral status because they are a person; the fetus does not yet have moral status because it is not yet a person.

(c) Gradualist accounts of moral status

The most popular view of moral status amongst the lay public is that the fetus gradually acquires increasing moral status during pregnancy, meaning that abortion at advanced gestations requires much stronger justification. This account will not support the Texas law. Even if one does accord some (or even full) moral status to the early fetus, this must be weighed against the interests and autonomy of the pregnant woman.^53^ As we have argued, the law represents a severe threat to both the health and autonomy of women.

The Texas law is a bare faced attempt to introduce anti‐abortion laws with a flimsy, or actually non‐existent, argument that will harm women, the health profession and society. It is problematic and unethical, and ought to be quashed.

## CONFLICT OF INTEREST

Julian Savulescu is a Partner Investigator on an Australian Research Council grant LP190100841 which involves industry partnership from Illumina. He does not personally receive any funds from Illumina. Julian Savulescu is a Bioethics Committee consultant for Bayer.

## Data Availability

N/A.
